# Supplementary material for: The GARP complex prevents sterol accumulation at the trans-Golgi network during dendrite remodeling
Source: J Cell Biol. 2022 Oct 14;222(1):e202112108. doi: 10.1083/jcb.202112108 (PMC9577387; doi:10.1083/jcb.202112108)
Supplement: Table S1 — lists primers used for generating and genotyping knockout flies by CRISPR. [file JCB_202112108_TableS1.docx]

**Table S1. Primers used for generating and genotyping knockout flies by CRISPR**

| *UAS plasmids* |  |
| --- | --- |
| Vps50 NotI 5’ | AAAAAGCGGCCGCATGCAGAATTCCAAGGCCAAAATGGA |
| Vps50 3x HA Kpn 3’ | AATTAGGTACCTCAGGTGGACCGGTGTCCGC |
| Vps53 5’ attB | GGGGACAAGTTTGTACAAAAAAGCAGGCTTACACCATGAGCGAGTCTGCG |
| Vps53 3’ attB | GGGGACCACTTTGTACAAGAAAGCTGGGTTGGGGAAGCGCTTTTTCAGTAGG |
| *CRISPR* |  |
| *Guide RNA sequences* | |
| Vps50 5’ guide RNA | CTTCGAGAAAATGCTAATCGGTACT |
| Vps50 3’ guide RNA | CTTCGGTACTTAAGTCATGGTGAA |
| Vps53 5’ guide RNA | CTTCGTAAATTGCTGTCGTGCTGTA |
| Vps53 3’ guide RNA | CTTCGATGTAGCCGTATATTAACT |
| Vps54 5’ guide RNA | CTTCGGCTGTTCGTTCTCTAGCCT |
| Vps54 3’ guide RNA | CTTCGCAATGAAGACGAACTAGGCT |
| *Primers for cloning homology arms* | |
| Vps50 5’ arm F | ATTCGCGGCCGCTGGAATGATGCCAGCTAGCAAAT |
| Vps50 5’ arm R | ATTCGCGGCCGCTTTAAGCACTTTTATACATATTCGTGGC |
| Vps50 3’arm F | AAAAACTAGTCCAAACCCTCCTTAAGTGCAA |
| Vps50 3’ arm R | AAAAACTAGTCGTTCGTCTGTCCACGTAGAG |
| Vps53 5’ arm F | AAAAGCGGCCGCAAAGTTATTGTTATTCTCTTCGTGG |
| Vps53 5’ arm R | AAAAGCGGCCGCTGTCATCACTGGCCGCTC |
| Vps53 3’arm F | AAAAACTAGTGACCTAATAAATTAAGCAACTAATCAT |
| Vps53 3’ arm R | TATAACTAGTGGAAAAGGATGTCTTTCATAGGTGAGT |
| Vps54 5’ arm F | ATTGCACCTGCCATGTCGCAGGGGAAAGTCTCCCGTCTTT |
| Vps54 5’ arm R | GTGTCACCTGCTAATCTACGCCATAACACATGCTCTGCG |
| Vps54 3’arm F | TTACGCTCTTCGTATGGCTCTACTGAGCATTCCGAA |
| Vps54 3’ arm R | CGTAGCTCTTCTGACTATGCGCACTCCAAATCCGTCCA |
| *Primers for genotyping* | |
| Vps50 WT F | ﻿TCTCCGACAACTGCTTTGCT |
| Vps50 WT R | ﻿CTCGTCCACAATGCCGGATA |
| Vps50 KO F | ﻿GGCTGCAGACGTTTTTGTGT |
| Vps50 KO R | ﻿CGGGAACTGCTCCAAGTTGA |
| Vps53 WT F | ﻿GAGTACGCCTCCAAAGTGCT |
| Vps53 WT R | ﻿GCATATCGCGCGTGAGTAAC |
| Vps53 KO F | ﻿CGGGTCCGAACAGTAACTCTC |
| Vps53 KO R | ﻿TAGGCGGATGGATTGCGAATA |
| Vps54 WT F | ﻿ACTTGAGTTCTGTGCCCGAG |
| Vps54 WT R | ﻿AGTGACTCAGCTGTTCCTGC |
| Vps54 KO F | ﻿GCCTAGAGAACGAACAGCCA |
| Vps54 KO R | ﻿ACCGCCATTGATTTGTACGC |
