# Supplementary material for: The GARP complex prevents sterol accumulation at the trans-Golgi network during dendrite remodeling
Source: J Cell Biol. 2022 Oct 14;222(1):e202112108. doi: 10.1083/jcb.202112108 (PMC9577387; doi:10.1083/jcb.202112108)
Supplement: Table S2 — shows additional statistics for Fig. 3 E. [file JCB_202112108_TableS2.docx]

**Table S2. Additional statistics for Fig3 E**

| **Tukey’s multiple comparisons test** | **Adjusted P Value** |
| --- | --- |
| 72hrs *+/+* vs *Vps50^KO/KO^* | >0.9999 |
| 72hrs *+/+* vs *Vps54^KO/KO^* | 0.4128 |
| 96hrs *+/+* vs *Vps50^KO/KO^* | 0.9588 |
| 96hrs *+/+* vs *Vps54^KO/KO^* | 0.0001 |
| 1 day *+/+* vs *Vps50^KO/KO^* | <0.0001 |
| 1 day *+/+* vs *Vps54^KO/KO^* | <0.0001 |
| 7 days *+/+* vs *Vps50^KO/KO^* | 0.0004 |
| 7 days *+/+* vs *Vps54^KO/KO^* | <0.0001 |
| 21 days *+/+* vs *Vps50^KO/KO^* | 0.0047 |
| 21 days *+/+* vs *Vps54^KO/KO^* | <0.0001 |
|  |  |
| *+/+* 72hrs vs 96hrs | <0.0001 |
| *+/+* 96hrs vs 1 day | 0.9423 |
| *+/+* 1 day vs 7 days | 0.0001 |
| +/+ 7 days vs 21 days | >0.9999 |
| *+/+* 72hrs vs 1 day | <0.0001 |
| *+/+* 72hrs vs 7 days | <0.0001 |
| *+/+* 72hrs vs 21 days | <0.0001 |
| *+/+* 96hrs vs 7 days | <0.0001 |
| *+/+* 96hrs vs 21 days | <0.0001 |
| *+/+* 1 day vs 21 days | <0.0001 |
|  |  |
| *Vps50^KO/KO^* 72hrs vs 96hrs | 0.0005 |
| *Vps50^KO/KO^* 96hrs vs 1 day | 0.2048 |
| *Vps50^KO/KO^* 1 day vs 7 days | <0.0001 |
| *Vps50^KO/KO^* 7 days vs 21 days | 0.9478 |
| *Vps50^KO/KO^* 72hrs vs 1 day | 0.8608 |
| *Vps50^KO/KO^* 72hrs vs 7 days | <0.0001 |
| *Vps50^KO/KO^* 72hrs vs 21 days | <0.0001 |
| *Vps50^KO/KO^* 96hrs vs 7 days | 0.1298 |
| *Vps50^KO/KO^* 96hrs vs 21 days | 0.0108 |
| *Vps50^KO/KO^* 1 day vs 21 days | <0.0001 |
|  |  |
| *Vps54^KO/KO^* 72hrs vs 96hrs | 0.0234 |
| *Vps54^KO/KO^* 96hrs vs 1 day | >0.9999 |
| *Vps54^KO/KO^* 1 day vs 7 days | 0.4085 |
| *Vps54^KO/KO^* 7 days vs 21 days | >0.9999 |
| *Vps54^KO/KO^* 72hrs vs 1 day | 0.02 |
| *Vps54^KO/KO^* 72hrs vs 7 days | <0.0001 |
| *Vps54^KO/KO^* 72hrs vs 21 days | <0.0001 |
| *Vps54^KO/KO^* 96hrs vs 7 days | 0.3802 |
| *Vps54^KO/KO^* 96hrs vs 21 days | 0.2573 |
| *Vps54^KO/KO^* 1 day vs 21 days | 0.2790 |
