# Supplementary figures and images for: The GARP complex prevents sterol accumulation at the trans-Golgi network during dendrite remodeling
Source: J Cell Biol. 2022 Oct 14;222(1):e202112108. doi: 10.1083/jcb.202112108 (PMC9577387; doi:10.1083/jcb.202112108)

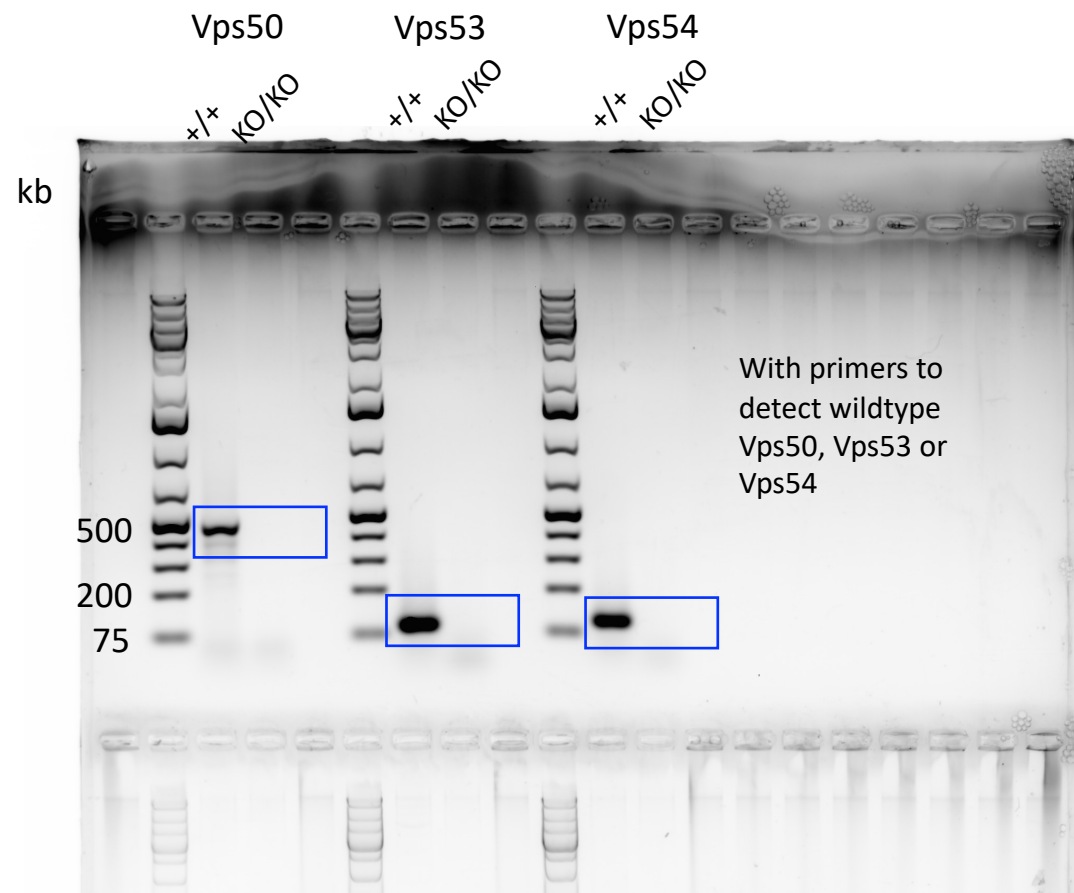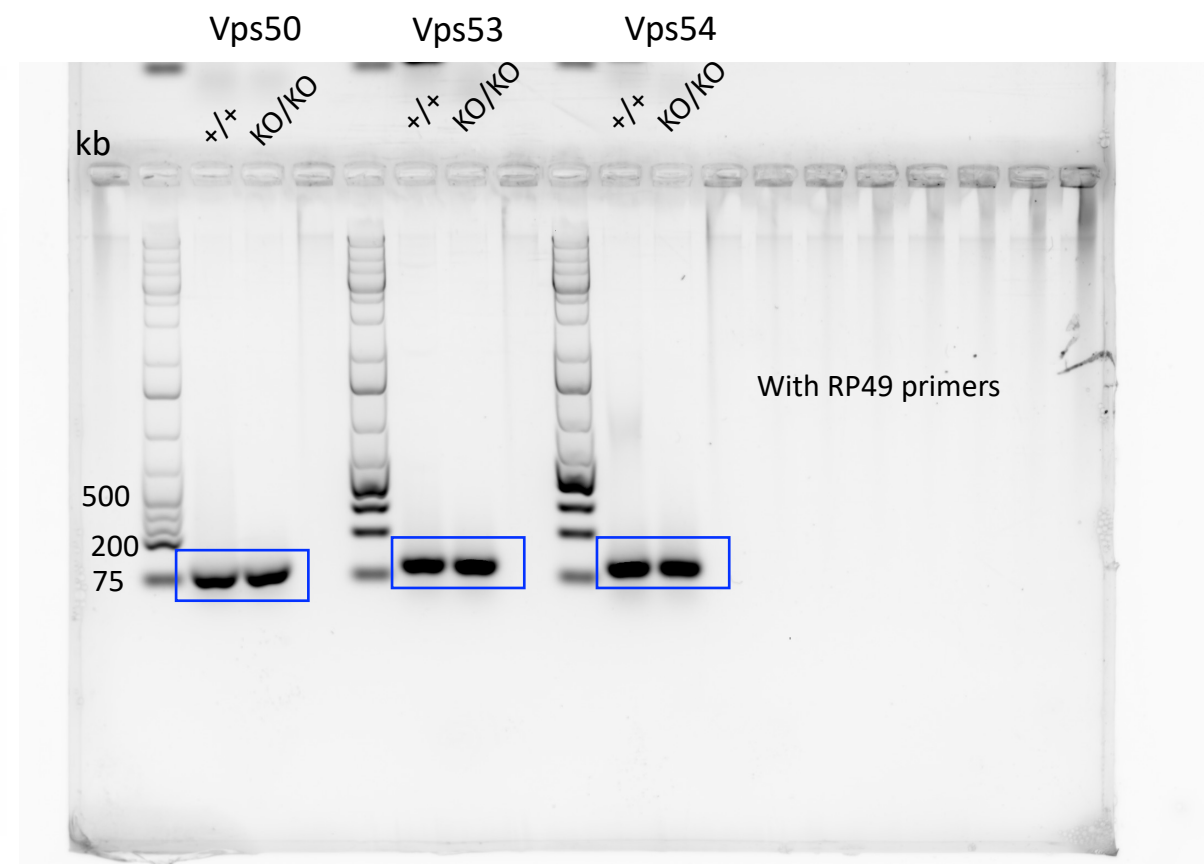

Supplement: SourceData F1 — is the source file for Fig. 1. [file JCB_202112108_SourceDataF1.pdf]

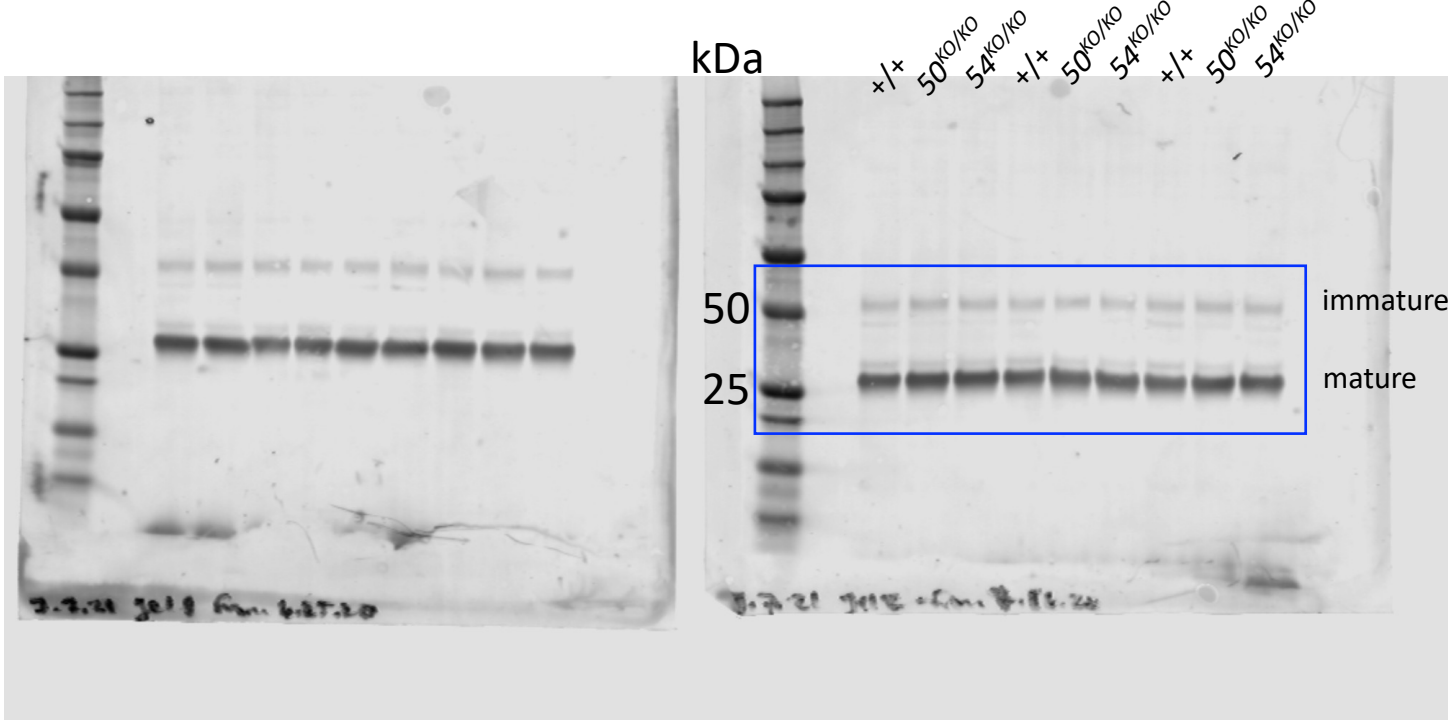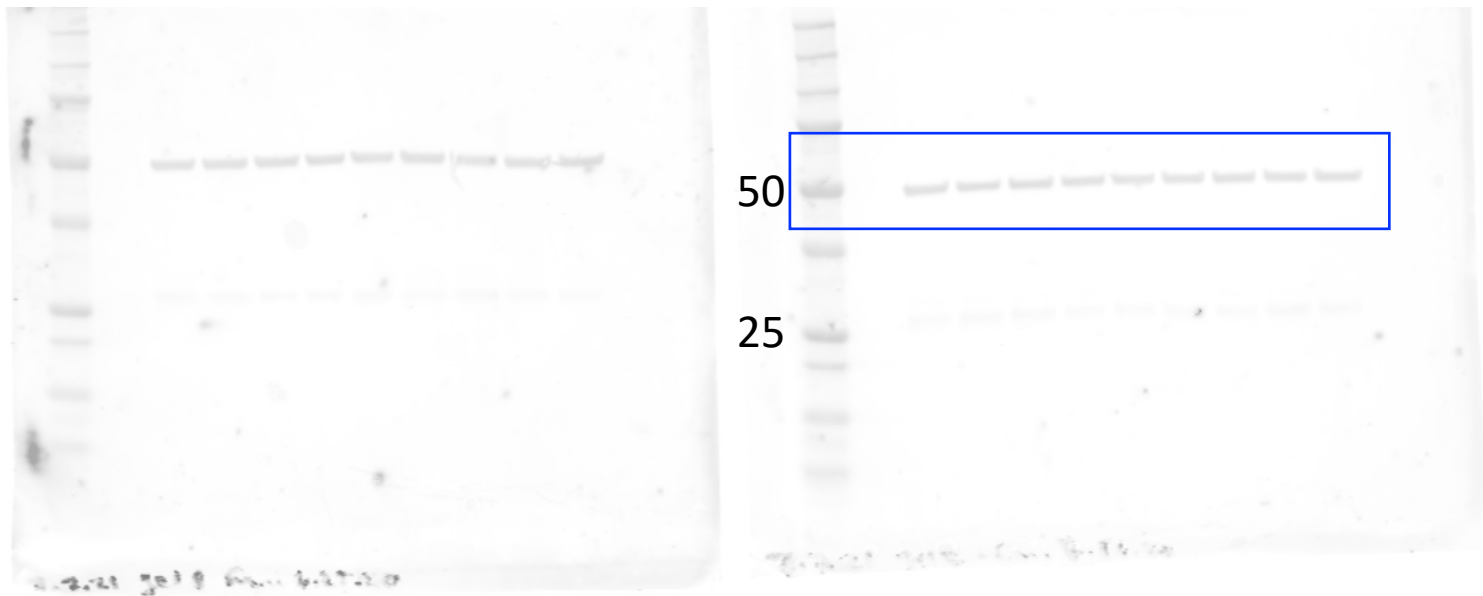

Supplement: SourceData FS3 — is the source file for Fig. S3. [file JCB_202112108_SourceDataFS3.pdf]
