# Supplementary material for: The GARP complex prevents sterol accumulation at the trans-Golgi network during dendrite remodeling
Source: J Cell Biol. 2022 Oct 14;222(1):e202112108. doi: 10.1083/jcb.202112108 (PMC9577387; doi:10.1083/jcb.202112108)
Supplement: SourceData FS1 — is the source file for Fig. S1. [file JCB_202112108_SourceDataFS1.pdf]

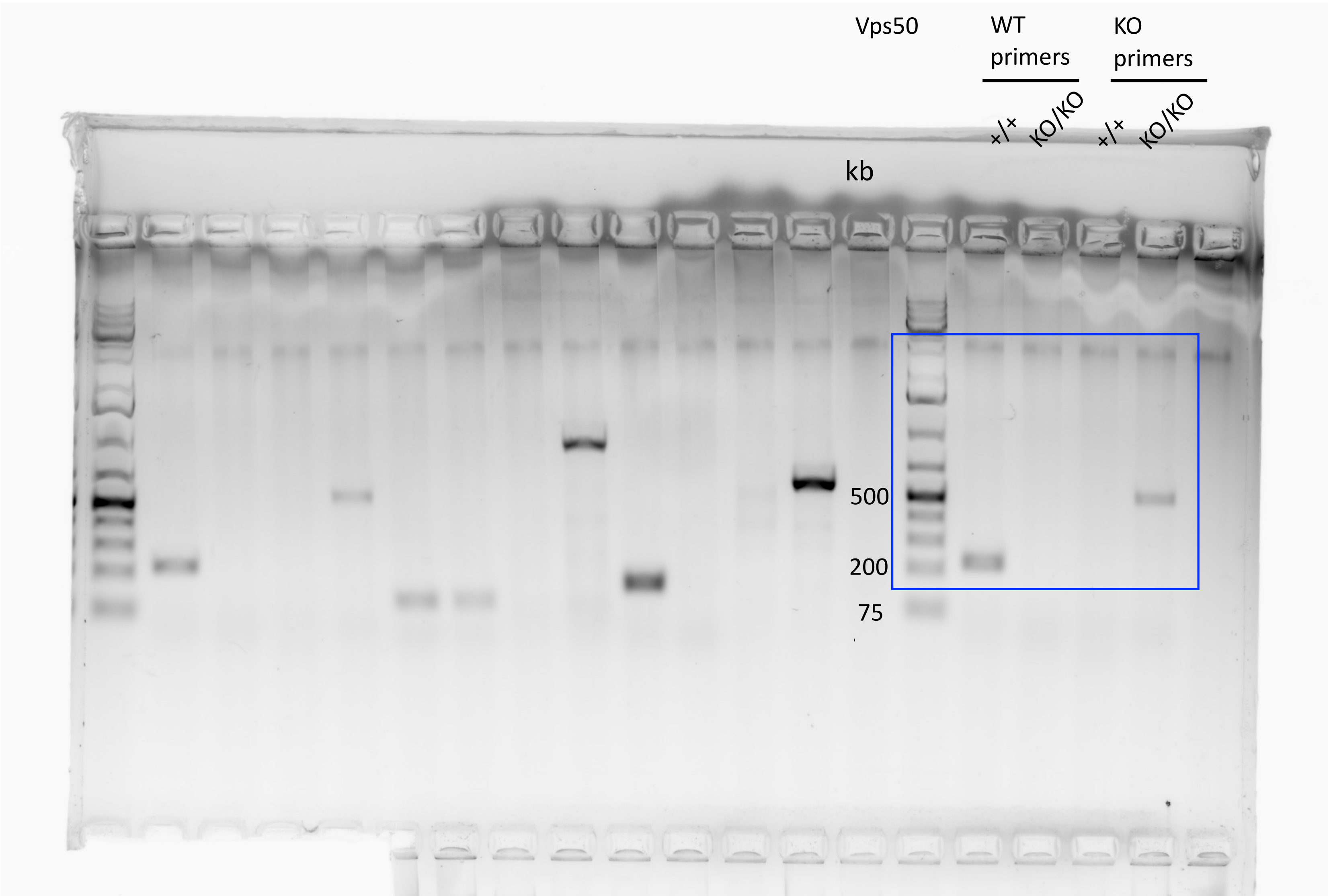

Vps50
WT primers
KO primers
+/+
KO/KO
+/+
KO/KO
kb
500
200
75

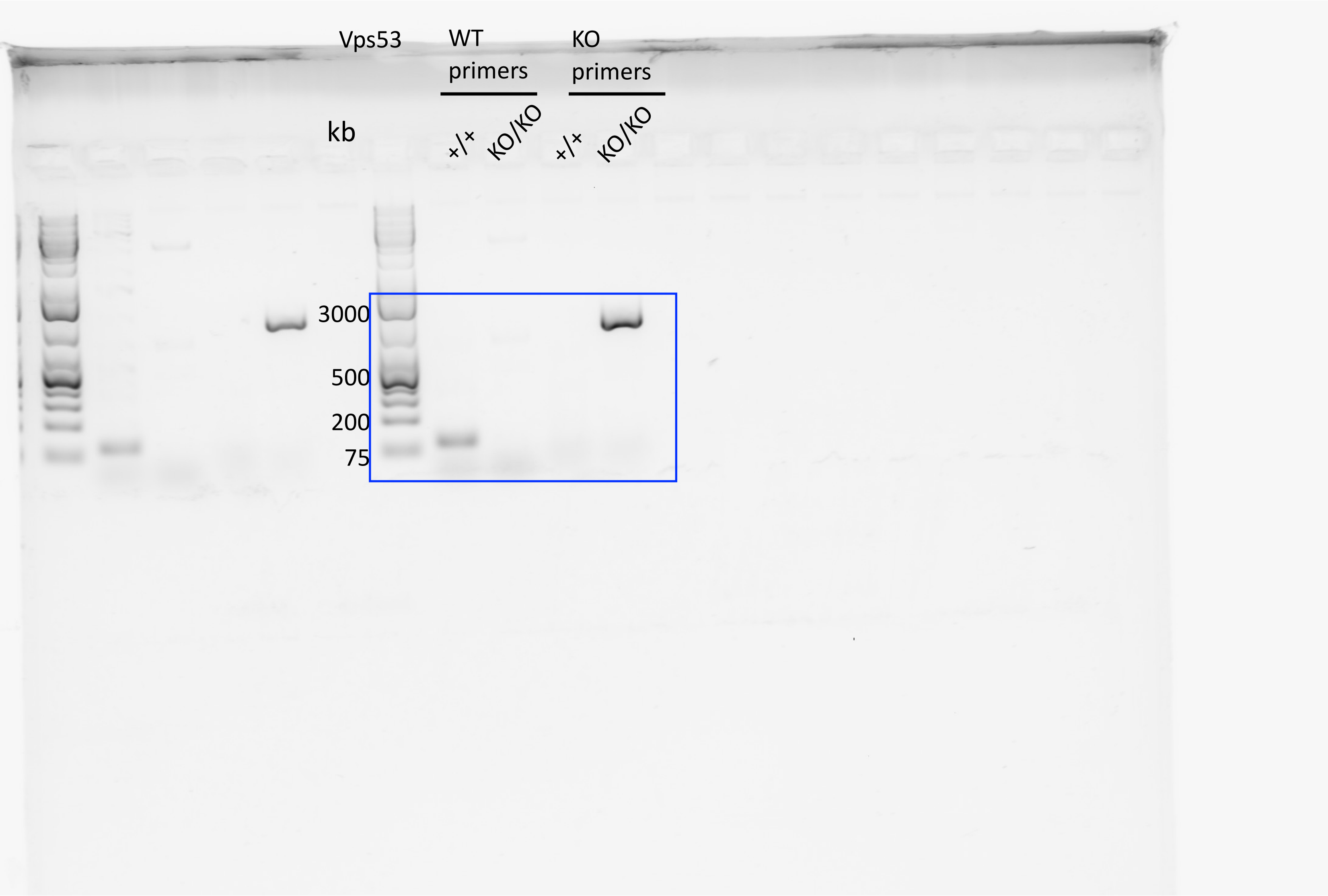
Vps53
WT primers
KO primers
kb
+/+
KO/KO
+/+
KO/KO
3000
500
200
75

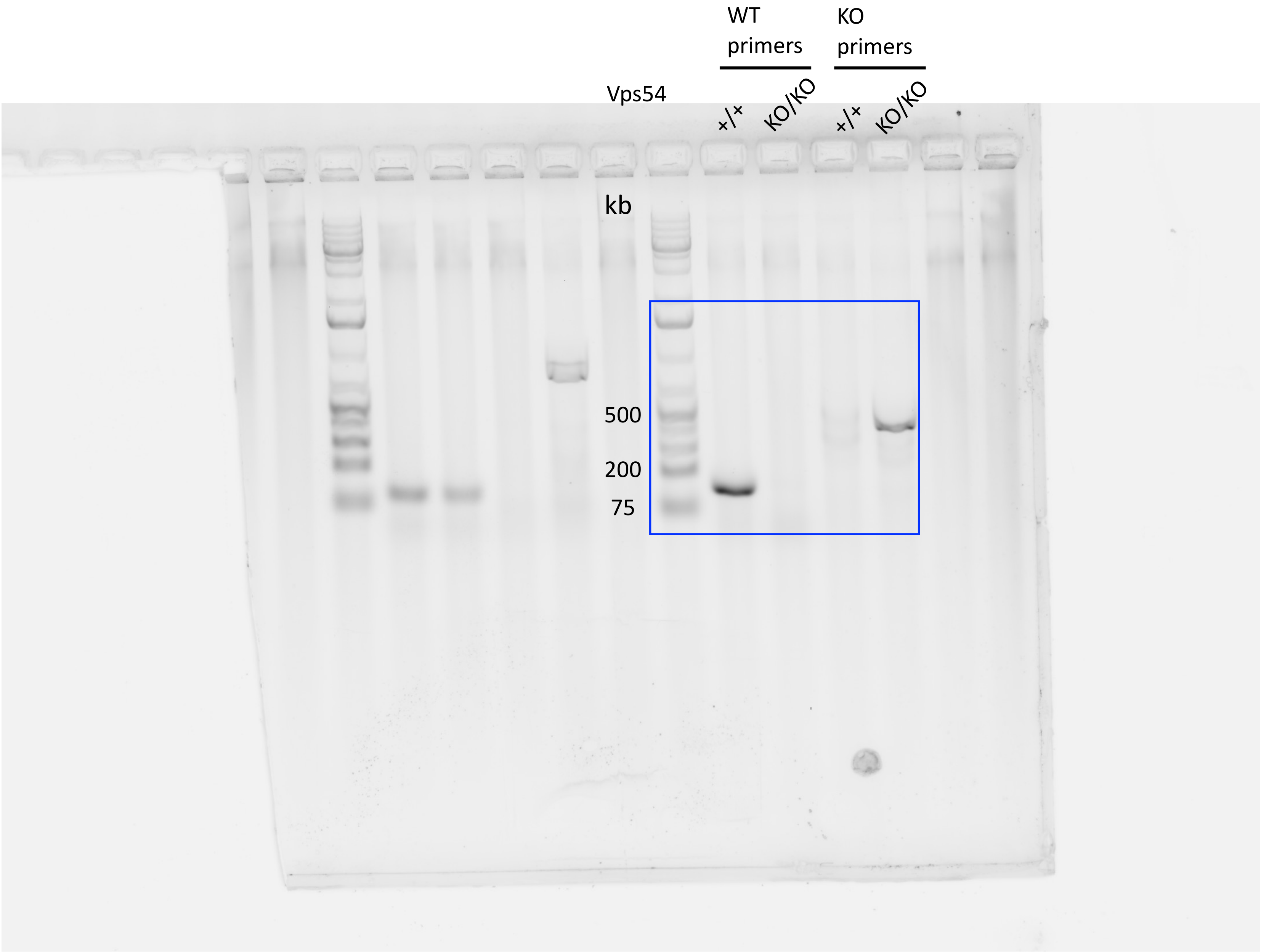
WT primers
KO primers
Vps54
+/+
KO/KO
+/+
KO/KO
kb
500
200
75

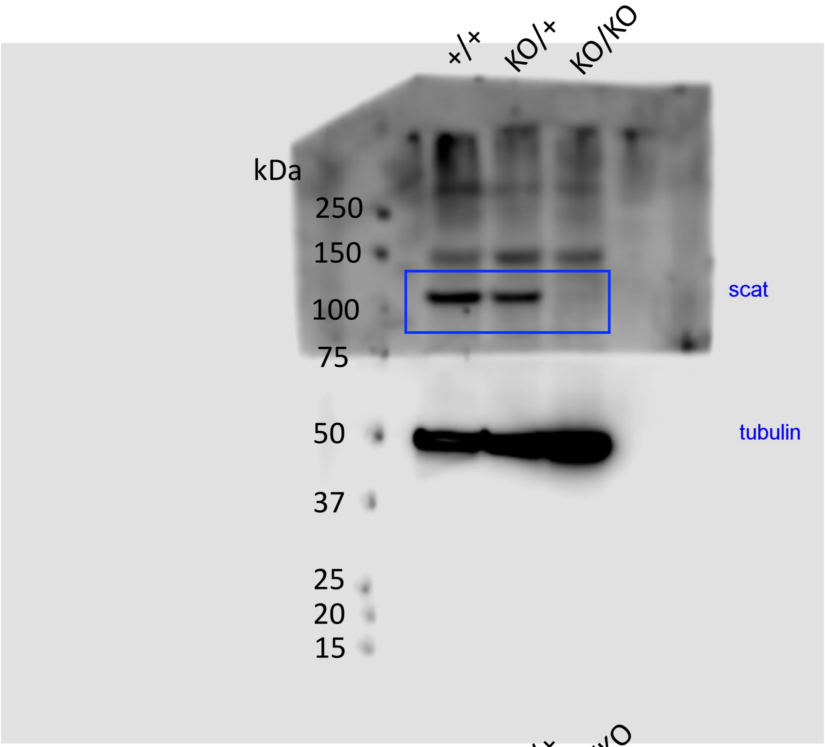

Membrane was cut at ~75kDa and the top was probed with anti-scat antibody, the bottom was probed with anti-tubulin antibody.

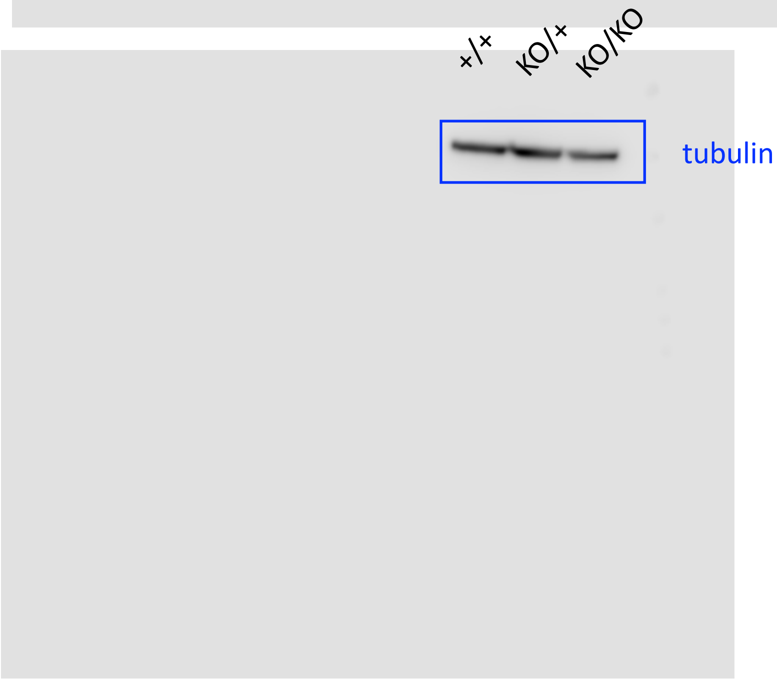

Because the original tubulin signal was so strong, we stripped the blot, incubated with a more diluted anti-tubulin solution and obtained a lighter exposure.
